# Decline in cardiorespiratory fitness in the Swedish working force between 1995 and 2017

**DOI:** 10.1111/sms.13328

**Published:** 2018-11-15

**Authors:** Elin Ekblom‐Bak, Örjan Ekblom, Gunnar Andersson, Peter Wallin, Jonas Söderling, Erik Hemmingsson, Björn Ekblom

**Affiliations:** ^1^ Åstrand Laboratory of Work Physiology The Swedish School of Sport and Health Sciences Stockholm Sweden; ^2^ Research Department HPI Health Profile Institute Danderyd Sweden; ^3^ Department of Medicine Karolinska Institutet, Karolinska University Hospital Solna Stockholm Sweden

**Keywords:** aerobic capacity, maximal oxygen consumption, population, secular trend, VO_2_max

## Abstract

**Background:**

Long‐term trend analyses of cardiorespiratory fitness (VO_2_max) in the general population are limited.

**Objectives:**

To describe trends in VO_2_max from 1995 to 2017 in the Swedish working force and to study developments across categories of sex, age, education, and geographic regions.

**Methods:**

A total of 354 277 participants (44% women, 18‐74 years) who participated in a nationwide occupational health service screening between 1995 and 2017 were included. Changes in standardized mean values of absolute (L/min) and relative (mL/min/kg) VO_2_max, and the proportion with low (<32) relative VO_2_max are reported. VO_2_max was estimated using a submaximal cycle test.

**Results:**

Absolute VO_2_max decreased by −6.7% (−0.19 L/min) in the total population. Relative VO_2_max decreased by −10.8% (−4.2 mL/min/kg) with approximately one‐third explained by a simultaneous increase in body mass. Decreases in absolute fitness were more pronounced in men vs women (8.7% vs 5.3%), in younger vs older (6.5% vs 2.3%), in short (11.4%) vs long (4.5%) education, and in rural vs urban regions (6.5% vs 3.5%), all *P* < 0.001. The proportions with low VO_2_max increased from 27% to 46% (*P* < 0.001).

**Conclusion:**

Between 1995 and 2017, there was a steady and pronounced decline in mean cardiorespiratory fitness in Swedish adults. Male gender, young age, short education, and living in a rural area were predictive of greater reductions. The proportion with low cardiorespiratory fitness almost doubled. Given the strong associations between cardiorespiratory fitness and multiple morbidities and mortality, preventing further decreases is a clear public health priority, especially for vulnerable groups.

## 1 INTRODUCTION

Low cardiorespiratory fitness (VO_2_max) is a strong independent predictor of poor metabolic health and increased risk for most non‐communicable diseases, as well as lower sustained, work productivity, and shorter life expectancy.^1^^,^ 2During recent decades, several behavioral and environmental factors have changed which may have negatively affected population levels of physical activity (PA) and thereby cardiorespiratory fitness., Together with an increased prevalence of overweight and obesity,^4^ it is plausible that the level of relative VO_2_max (mL/min/kg) has decreased. However, previous studies of secular trends in VO_2_max are limited to military conscripts^5^, ^6^, ^7^ or smaller samples of the general population,^8^, ^9^, ^10^ meaning that there is a lack of studies on secular trends in large populations of adults. Women are understudied, and with the alarming inequality in health and longevity between socioeconomic groups^11^ and an expected significant increase in multi‐morbidity among the older population over the next decades,^12^ subgroups analyses are highly clinically relevant.

Health Profile Assessment (HPA) has been carried out in occupational health services in Sweden for almost 40 years to promote health, collecting data from approximately 40 000 annual examinations during the last years.^13^ The combination of the large amount of HPA performed each year and the long‐term use of established and standardized methods in occupational health promotion generates a unique database, which enables analyses of level of and change in estimated VO_2_max in the Swedish working population over several decades.

The primary aim of this paper was to describe secular trends in estimated VO_2_max from a submaximal cycle ergometer test between 1995 and 2017 in a large sample of the working Swedish population, aged 18 to 74 years, and to study potential variations between women and men, different age‐groups, educational levels, and regions.

## 2 MATERIALS AND METHODS

This study was based on cohort data from the HPA database, managed by the HPI Health Profile Institute (Stockholm, Sweden), which also is responsible for standardization of methods used and education of the HPA coaches since inception. The HPA is an interdisciplinary method^13^, ^14^ and includes an extensive questionnaire, measurements of anthropometrics and blood pressure, a submaximal cycle test for estimation of VO_2_max and a person‐centered dialogue with an HPA coach. Participation is voluntary, is free of charge, and is offered to all employees working for a company or organization connected to occupational or other health service. From October 1982 until May 2017, 437 676 participants (18 to 74 year old) with a first‐time HPA and providing data on gender, age, and educational level were stored in the central database. The annual rate of participants was substantially lower in the first years, 1982 (n = 1) and 1994 (n = 888), compared to the following full years, 1995 (n = 1 347) vs 2016 (n = 31 529). To minimize influence of uncertainty and variations in the data collection procedure, we limited our analyses to 1995‐2017 (n = 436 126). Of these, 81.2% (n = 354 277) provided valid data of estimated VO_2_max and were included in the analyses. All participants provided informed consent prior to data collection. The study was approved by the ethics board at the Stockholm Ethics Review Board (Dnr 2015/1864‐31/2 and 2016/9‐32), and adhered to the Declaration of Helsinki.

### 2.1 Estimation of VO_2_max

Measurement of actual VO_2_max by a graded test to exhaustion in the general population is limited by numerous factors, including health risks in non‐athletic population and dependence on laboratory equipment and expertise. Therefore, VO_2_max was estimated from the standardized Astrand submaximal cycle ergometer test.^15^ Criterion validity has been tested for the Astrand test, showing no systematic bias and limited variation in mean difference between estimated and directly measured VO_2_max, mean difference 0.01 L O_2_/min (95% CI −0.10 to 0.11).^8^, ^16^ All participants were requested to refrain from vigorous activity the day before the test, consuming a heavy meal 3 hours and smoking/snuff use 1 hour before the test, and avoiding stress. The participant cycled on a calibrated ergometer at an individually adapted submaximal work rate for 6 minutes to achieve a steady‐state pulse. Using the steady‐state pulse, VO_2_max was estimated from a sex‐specific nomogram, with corresponding age‐correction factors, expressed as absolute (L/min) and relative (mL/min/kg) VO_2_max.

### 2.2 Other measurements

Body mass was assessed with a calibrated scale in lightweight clothing to the nearest 0.5 kg. Body height was measured to the nearest 0.5 cm using a wall‐mounted stadiometer. Highest educational attainment and place of dwelling (as county in Sweden of residence) at the time for the HPA was obtained by linking the personal identity number of the participants with data from Statistics Sweden.

### 2.3 Internal dropout analysis

Out of the total study population with a HPA since 1995, 81 849 participants (18.8%) lacked data on estimated VO_2_max. Reasons for a non‐valid VO_2_max were medication affecting the heart rate (such as betablockers) or heart rate outside the valid range. Some participants could not perform the test because of pain complaints, illness or perceived inability. Internal participation analyses for each 2‐year time period between 1995 and 2017 revealed that included participants, compared to excluded participants, were younger (42.2 vs 46.0 years, *P* < 0.001), had lower body mass (78.1 vs 81.4 kg, *P* < 0.001) and had higher education (27.9% university degree vs 22.8%, *P* < 0.001); however, the differences were generally small (Table S1).

### 2.4 Statistical analysis

For analyses of change in VO_2_max between 1995 and 2017, years were grouped into 2‐year periods (except the first period where we used 3 years) for reducing variations between years and for increasing statistical power. Mean values of estimated absolute and relative VO_2_max per 2‐year period were standardized, using the direct method, to the population 18‐74 years old in Sweden in 2015 (n = 6,842,976) by sex, age (18‐24 years, 25‐34 years, 35‐44 years, 45‐49 years, 50‐54 years, 55‐64 years, 65‐74 years), and length of education (<9 years; 10‐12 years; ≥12 years). Standardized mean values were calculated in order to account for yearly variations in important prognostic variables (age, education, gender, and region). Standardized mean values were stratified by sex, age (18‐34 years, 35‐49 years, 50‐74 years), education (<9 years, 10‐12 years, ≥12 years), and county (counties categorized as including the three largest cities of Sweden “Urban,” counties including a majority of rural municipalities defined by Swedish Association of Local Authorities and Regions “Rural,” and all other counties “All other”). Linear regression models were applied to study changes in absolute and relative VO_2_max over the study period within the total population and across subgroups. Absolute and relative VO_2_max, respectively, were introduced as dependent variable, and sex, age, educational level, region, and year performed as independent variables. Significant change was defined as *P* < 0.05 for the performed year variable. To study the interaction between subgroups in decrease of absolute and relative VO_2_max, an interaction term (performed year*sub‐group) was introduced in the above regression analyses. Significant interaction(s) were defined as *P* < 0.05 for the interaction term. As all changes and interaction analyses were significant, statement of a decrease or interaction in the manuscript refers to a significant decrease or interaction. To study the change in absolute and relative VO_2_max per year between different subgroups, the probability values were computed for the difference between the B‐coefficients.^17^ Proportions of women and men with low relative VO_2_max (<32 mL/min/kg^18^) per 2‐year period were calculated and standardized, using the direct method, to the same population as for the mean values (above). For sensitivity analyses, lower cutoffs, by 1 MET steps (3.5 mL/min/kg), were also analyzed; <28.5, <25, and <21.5 mL. Sex‐specific odds ratios (95% CI), adjusted for age and education level, were obtained to study and compare the annual change in proportion below each cutoff. Levene's test for equality of variances was used to study potential increased variance within subgroups between the first five and last 5 years of the study period. The statistical analyses were conducted using IBM SPSS (Statistical Package for the Social Sciences for Windows), version 24.0.0, 2016, SPSS Inc, Chicago, IL and SAS version 9.4.

## 3 RESULTS

Participation rates by age‐group (18‐34, 35‐49 and 50‐74 years) were similar over time, while a variation in proportion of men and women as well as participants with high education from 1995 to 2017 was more pronounced (Table 1). Standardized mean body mass was higher in both men (4.2%) and women (4.8%) in the latter compared with the early years.

**Table 1:** Distribution of sex, age, and educational level as well as standardized mean (SD) of height (cm) and weight (kg) in the study population, 1995‐2017

Year										Women			Men		
	N	Sex		Age			Years of education			n	Height Mean (SD)	Weight Mean (SD)	n	Height Mean (SD)	Weight Mean (SD)
		Women	Men	18‐34 y	35‐49 y	50‐74 y	≤9 y	10‐12 y	>12 y						
1995‐1997	4574	52%	48%	30%	48%	22%	16%	70%	14%	2395	165.4 (0.5)	66.2 (0.7)	2179	179.9 (0.4)	82.4 (0.4)
1998‐1999	6543	45%	55%	28%	44%	28%	13%	67%	19%	2964	166.4 (0.4)	66.7 (0.6)	3579	179.6 (0.4)	82.8 (0.6)
2000‐2001	12 545	49%	51%	28%	42%	31%	12%	67%	21%	6206	166.6 (0.3)	67.5 (0.6)	6339	180.0 (0.3)	84.5 (1.2)
2002‐2003	22 629	52%	48%	29%	42%	29%	11%	69%	20%	11 858	166.7 (0.5)	67.3 (0.4)	10 771	179.7 (0.2)	83.4 (0.5)
2004‐2005	37 420	52%	48%	26%	44%	31%	10%	65%	25%	19 500	166.2 (0.3)	68.4 (0.6)	17 920	179.3 (0.4)	82.6 (0.7)
2006‐2007	38 519	49%	51%	25%	44%	31%	10%	65%	25%	18 714	166.2 (0.3)	68.4 (0.4)	19 805	179.8 (0.3)	83.9 (0.6)
2008‐2009	43 479	46%	54%	26%	43%	31%	10%	65%	26%	20 068	166.2 (0.3)	68.8 (0.4)	23 411	179.7 (0.3)	84.5 (0.6)
2010‐2011	39 177	44%	56%	26%	45%	29%	9%	63%	27%	17 301	166.3 (0.2)	69.6 (0.4)	21 876	180.1 (0.3)	85.1 (0.6)
2012‐2013	57 246	41%	59%	27%	45%	28%	8%	61%	31%	23 336	166.6 (0.2)	69.5 (0.5)	33 910	180.0 (0.3)	85.0 (0.6)
2014‐2015	55 584	38%	62%	30%	43%	28%	7%	63%	30%	20 894	166.3 (0.3)	69.8 (0.5)	34 690	179.9 (0.3)	85.5 (0.6)
2016‐2017	36 561	37%	63%	33%	40%	27%	7%	64%	29%	13 464	166.1 (0.3)	69.4 (0.5)	23 097	179.9 (0.3)	85.9 (0.6)
Total	354 277	44%	56%	28%	43%	29%	9%	64%	27%	156 700	166.3 (0.3)	68.3 (0.6)	197 577	179.8 (0.3)	84.1 (0.7)

**Figure 1:**
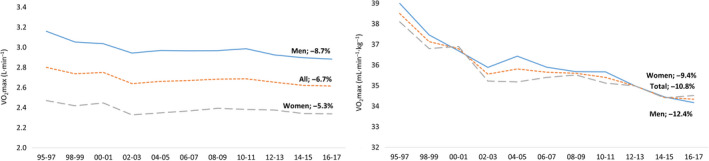
Change in standardized mean of absolute (L/min, left) and relative (mL/min/kg, right) VO_2_max from 1995 to 2017 in the total study sample and in relation to sex

Absolute VO_2_max decreased by 6.7% (−0.19 L/min) in the total population between 1995‐1997 and 2016‐2017 (Figure 1, Table S2). Men had higher levels of absolute VO_2_max and experienced a greater decrease compared to women; −8.7% (−0.28 L/min) vs −5.3% (−0.13 L/min).

Relative VO_2_max decreased even more in the total population (−10.8%, −4.2 mL/min/kg), in men (−12.4%, −4.8 mL) and women (−9.4%, −3.6 mL) (Figure 1, Table S2). The decrease in relative VO_2_max was, to one‐third, explained by a simultaneous increase in body mass.

**Figure 2:**
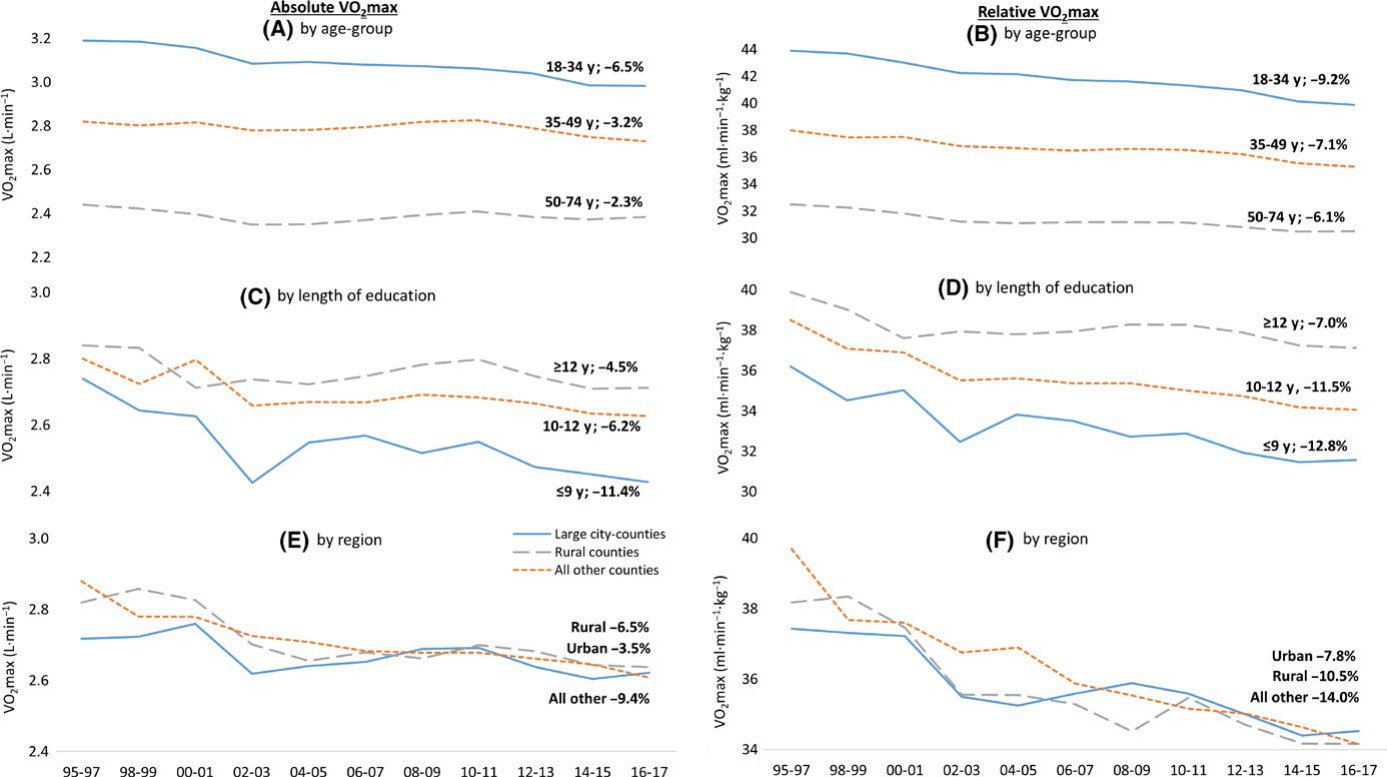
Change in standardized mean of absolute (L/min, left) and relative (mL/min/kg, right) VO_2_max from 1995 to 2017 in relation to age‐group (A and B), length of education (C and D), and region (E and F)

Younger age‐groups had higher absolute and relative VO_2_max compared to middle‐aged and older age‐groups (Figure 2 A,B, Table S3). Decreases were most pronounced in the youngest age‐group (absolute VO_2_max −6.5%, relative VO_2_max −9.2%), compared to the middle (−3.2% and −7.1%) and oldest age‐group (−2.3% and −6.1%). This was seen for both men and women (Table S6); however, the differences in decrease for relative VO_2_max were similar in all male age‐groups due to a larger increase in body mass in the middle‐aged and older age‐groups.

Participants with shorter education had lower absolute and relative VO_2_max throughout the whole study period compared to participants with longer education (Figure 2 C,D, Tables S4 and S7). The decrease in absolute VO_2_max was greater in participants with short (−11.4%) compared to medium (−6.2%) and long (−4.5%) education. A simultaneous increase in body mass resulted in a greater decrease in relative VO_2_max (−12.8%, −11.5%, and −7.0%, respectively). Participants with ≥12 years of education experienced a levelling‐off in the decrease over the first 10 years of the 21st century. While the reductions were similar across all age‐groups in participants with short and medium educational attainment, only the youngest age‐group experienced a significant decrease in VO_2_max in participants with high education (Table S8).

There was a decrease in absolute and relative VO_2_max in all county‐groups (Figure 2 E,F, Table S5). Starting off with a higher value in 1995‐1997, both the rural county group (absolute VO_2_max −6.5%, relative VO_2_max −10.5%) and all other counties (−9.4% and −14.0%) had a steeper decrease in VO_2_max compared to the group with large city‐counties (−3.5% and −7.8%). However, all county‐groups had similar values at the end of the study period. Participants with long education and in counties including the three largest cities had a lower yearly decrease in relative VO_2_max compared to participants in lower educational levels and other counties, respectively (Table 2).

**Table 2:** Changes in cardiorespiratory fitness per year in the total population and across subgroups

	Absolute VO_2_max (ml·min^−1^)	Relative VO_2_max (ml·min^−1^·kg^−1^)
	B (95% CI)	B (95% CI)
Total	−6.8 (−7.2 to −6.4)	−0.13 (−0.14 to −0.13)
Women	−2.4 (−3.0 to −1.9)^a^	−0.09 (−0.09 to −0.08)^a^
Men	−10.0 (−10.6 to −9.4)	−0.17 (−0.18 to −0.17)
Age^b^		
18–34 y	−14.2 (−15.1 to −13.3)	−0.24 (−0.25 to −0.22)
35–49 y	−5.4 (−6.0 to −4.8)	−0.12 (−0.13 to −0.11)
50–74 y	−0.4 (−1.1 to 0.3)	−0.06 (−0.07 to −0.05)
Length of education		
≤9 y	−8.7 (−10.0 to −7.5)	−0.14 (−0.16 to −0.12)
10–12 y	−7.5 (−8.0 to −7.0)	−0.15 (−0.16 to −0.15)
≥12 y	−3.8 (−4.7 to −2.9)^c^	−0.08 (−0.09 to −0.07)^c^
Region (counties)		
Urban	−5.8 (−6.5 to −5.1)^d^	−0.11 (−0.12 to −0.10)^d^
Rural	−7.5 (−8.4 to −6.6)	−0.16 (−0.17 to −0.14)
All other	−7.9 (−8.6 to −7.2)	−0.16 (−0.17 to −0.15)

Values are adjusted for sex, age, education level and weight (only relative VO_2_max values).

a Significantly different women vs. men.

b Significantly different between all age‐groups.

c Significantly different from ≤9 years and 10–12 years.

d Significantly different from rural and all other counties.

The yearly decrease over the study period was 6.8 mL/min and 0.13 mL/min/kg, respectively, with a steeper annual decrease in relative VO_2_max at the end of the 1990 s and 2010 to 2017 compared to the first decade of the 21st century (Table 2). Men experienced a greater decrease in relative VO_2_max per year compared to women, as well as younger age‐groups compared to older.

**Figure 3:**
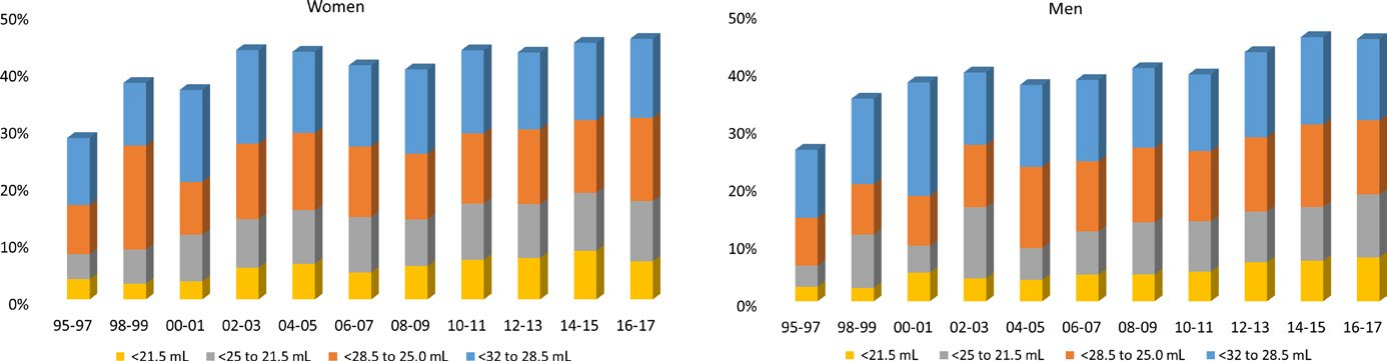
Standardized proportions of women (left) and men (right) with a low VO_2_max using different cutoffs, from 1995 to 2017

The proportion with low VO_2_max (<32 mL/min/kg) increased significantly over the study period, from 27% in 1995‐1997% to 46% in 2016‐2017, with a small but significantly greater increase in men (26% to 46%) compared to women (28% to 46%), *P* < 0.001 (Figure 3, Table S9). Proportions below each lower cutoff (<28.5, 25, 21.5 mL) increased even further (*P* < 0.001) in both men and women.

Potential change in variance in relative VO_2_max between the first five (1995‐1999) and the last five (2013‐2017) years of the study period is presented in Table S10. The variance was greater in participants with long education, among middle‐aged and older at the end of the study period, while the variance was smaller in young women with short education.

## 4 DISCUSSION

In this large cohort with data spanning from 1995 to 2017, we found evidence of a consistent and considerable decrease in absolute cardiorespiratory fitness (VO_2_max) of −6.7% (−0.19 L/min) in a large sample of Swedish adults. The decrease in relative cardiorespiratory fitness was even more pronounced, −10.8% (−4.2 mL/min/kg), only partly explained by a simultaneous increase in body weight. In sub‐group analyses, we found that reductions were more pronounced in men, in young age‐groups, in those with short education, and in rural regions. The proportions with low cardiorespiratory fitness (<32 mL) increased substantially over the study period, from 27% to 46% in the total study population, with greater, relative increases using lower cutoffs.

The present findings are similar to previous studies in smaller population samples and young, male military conscripts. Craig et al reported a lower relative VO_2_max in 2007‐2009 compared to 1981 in Canadian children and adults.^10^ Repeated population‐based cross‐sectional studies in Swedish adults showed no change in absolute or relative VO_2_max in women between 1990, 2001 and 2013.^8^, ^9^ But a decrease in relative VO_2_max in younger and middle‐aged men between 1990 and 2001, and in the total male group between 1990 and 2013. The decrease in relative VO_2_max was mainly due to an increase in body mass. In male Swedish military conscripts, no change was seen in maximal working capacity (absolute VO_2_max) assessed by cycle ergometer between 1986 and 1995, however, with mean increase in body mass of 1.9 kg over the study period.^5^ Moreover, relative VO_2_max was lower in Norwegian 18‐year‐old men in 2002 compared to 1980,^6^ and distance achieved in a 12‐minutes running test decreased with almost 400 m between 1980 and 2015 in Finnish male conscripts.^7^ The decline in performance in the two latter cohorts was mainly explained by a simultaneous increase in body mass. The discrepancy between previous studies and the present study of change in absolute VO_2_max is highly interesting and may partly be due to the different population studied. Though, from a public health point of view, the present result is alarming and may have an even greater impact on the health panorama, as a lower absolute aerobic work capacity as well as a higher body mass both have an independent association with increased disease risk and reduced longevity.^18^, ^19^

Albeit a shift in behavioral and environmental factors potentially decreasing the levels of vigorous PA in the general population, secular trend analyses of leisure‐time PA, including sports participation, show increasing levels during the past 30 years in the adult population in high‐income countries.^8^, ^20^, ^21^ The proportion of Swedish adults reporting high‐intensity exercise ≥two times/wk has increased, in all age‐groups and in all levels of education, between the late 1980 s and 2006‐2007.^22^ This level of exertion should be sufficient for at least maintaining level of VO_2_max in these subjects. However, whether self‐reported higher levels of intense activity reflect an actual increase in high‐intensity exercise can be questioned. Although an increased participation rate between 1993 and 2007 in the world's largest cross‐country race held annually in Sweden, increased run times were seen in both top, mean, and bottom quartiles, as well as in the top and bottom 5%, irrespectively of sex and age.^23^ However, during the same time period, work‐related PA has decreased significantly, with a shift from occupations requiring moderate‐to‐vigorous PA to predominantly sedentary or light PA occupations.^20^, ^21^, ^24^ As sufficient amount of physical stress of the cardiorespiratory system is required to maintain or increase VO_2_max, it could be hypothesized that the lower work‐related levels of more intense PA may partly explain the decrease of VO_2_max in the studied population of Swedish employees and may be a target area for future interventions.

One sub‐group that exhibited no or low decrease in both absolute and relative VO_2_max was middle‐aged and older participants with long education, especially during the first 10 years of the 21st century. Looking at potential time trends of participation in events requiring more strenuous physical activity, there was an explosion in numbers of marathon finishers in Europe around the turn of the millennium, with approximately 200 000 finishers in year 2000 to 600 000 in 2011.^25^ Running is an easy accessible form of exercise, however, also with a strong gradient in relation to educational level; those with highest education engages to a greater extent to endurance and strenuous exercise than those with lower education.^25^ Trend data from Statistics Sweden also reveal an accelerating proportion of the population that around the turn of the millennium reports high‐intensity exercise at least two times a week, with a more pronounced increase in middle‐aged and older adults but similar across educational levels.^22^ Although highly speculative, the increased interest and participation rates in strenuous forms of activity in some subgroups of the population may have had an impact on the lower decline of VO_2_max in these sub‐populations. However, the increased intra‐individual variance between the early and the latter years of the study period in the same subgroups may also indicate that a possible increase in participation in more strenuous activity may be limited to a part of, rather than the full, population of the sub‐group.

The mean decrease in VO_2_max of 4.2 mL/min/kg, with even larger decreases in some subgroups, is highly clinically relevant. A 1 MET (3.5 mL/min/kg) increase in VO_2_max has been associated with 13% and 15% decreased risk of all‐cause mortality and CVD events, respectively.^26^ Moreover, a 1 MET improvement in fitness between baseline and a second examination was associated with a 7%, 22%, and 12% lower risk of subsequent incidence of hypertension, metabolic syndrome, and hypercholesterolemia, respectively, after 6‐year follow‐up in healthy adults.^19^

Moreover, the considerable increase in proportion of both women and men with low fitness level is notable. Low fitness level has previously been linked to a substantially higher risk of all‐cause mortality after 8‐year follow‐up.^27^ The population‐attributable risks assessed in the same study revealed that 9% and 15% of all deaths in men and women, respectively, with low VO_2_max in the studied population might have been prevented if they had become more fit. Halting the gradual reduction in cardiorespiratory fitness is a clear public health priority for a sustainable future and of high clinical relevance, mainly by providing improved opportunities for regular physical activity. The greater decline in specific subgroups, with increasing gaps between subgroups, is especially alarming and may be primary targets for interventions to improve health in this population. For example, the steeper decrease in participants with short education is alarming. Lower educational level or socioeconomic status compared to higher has previously been associated with lower VO_2_max and/or fulfillment of recommended levels of moderate‐to‐vigorous PA.^28^, ^29^ This is suggested as one important contributing factor to the social inequality in health between socioeconomic groups, with low socioeconomic status associated with higher burden of disease and shorter life expectancy.^11^

The main strength of the present study is the large sample with yearly assessments of VO_2_max in the Swedish working population over a 23‐year period, with the potential to perform highly clinically relevant analyses of variations across subgroups. Previous elucidation of change in cardiorespiratory fitness over several decades in a large population‐based sample is, to our knowledge, non‐existing. Standardization of data in relation to the Swedish population with regard to sex, age, and length of education enabled comparison over the study period. A limitation was relatively lower number of participants during the early years compared to the latter, inducing a lower power. Another limitation is the use of a submaximal test to estimate VO_2_max. However, measuring actual VO_2_max during maximal performance would not have been feasible in this large non‐athletic population, together with HPA test leaders not experts in work physiology nor with access to laboratory equipment. In addition, assessment of VO_2_max by the Astrand protocol is reported to yield a valid and reliable estimation of actual VO_2_max.^8^, ^16^ Participants on medication that could affect heart rate response during the submaximal test were not excluded in the present study. In the total study population, 79% of the participants reported no medication, 3% reported medication, and 18% lacked data on medication use. The relative proportion of participants reporting medication or with missing data (with the possibility that they were on medication) increased over the years from 1995 to 2017. However, most cardio‐protective treatments, including anti‐hypertensive medication, have typically heart rate‐lowering effects, which in turn would yield a higher estimated VO_2_max during the submaximal cycle test. So, if anything, the decline in estimated VO_2_max over the years could be somewhat underestimated. We had only data on county of residence and not on municipality of residence. This might have yielded a too rough classification of the population when analyzing potential region differences.

## 5 PERSPECTIVE

The present study provides for the first time evidence of an overall deterioration in cardiorespiratory fitness in a large cohort of men and women over the last three decades, which may have had a negative impact on performance and health in this population. Previous studies of secular trends in VO_2_max have been limited to military conscripts or smaller samples of the general population. The main driver for the decline was a deterioration in cardiorespiratory aerobic capacity, and only partly by a simultaneous increase in body mass. The reduction was particularly pronounced in men, in younger ages, in participants with a low educational level, and in rural regions. Given the strong associations between cardiorespiratory fitness and multiple morbidities and mortality, preventing further decreases is a clear public health priority, especially for vulnerable groups. Replication of findings in other countries and populations are needed.

## CONFLICT OF INTEREST

GA (responsible for research and method) and PW (CEO and responsible for research and method) are employed at HPI Health Profile Institute. JS reports personal fees from HPI Health Profile Institute during the conduct of the study.
