# Supplementary material for: Decline in cardiorespiratory fitness in the Swedish working force between 1995 and 2017
Source: Scand J Med Sci Sports. 2018 Nov 15;29(2):232–9. doi: 10.1111/sms.13328 (PMC7379642; doi:10.1111/sms.13328)
Supplement: Supplementary file 1 [file SMS-29-232-s001.pdf]

Supplement Table 1. Internal drop-out analyses within the available database, comparing individuals with (included) and without (excluded) VO_2max.

	Gender				Age		Height		Weight		>12 years of education	
	Included		Excluded		Included	Excluded	Included	Excluded	Included	Excluded	Included	Excluded
Year	n	% women	n	% women	Mean (SD)	Mean (SD)	Mean (SE)[#]	Mean (SE)[#]	Mean (SE)[#]	Mean (SE)[#]	%	%
1995-1997	4 574	52.4	815	41.6[a]	40.9 (10.0)	43.3 (10.6)[a]	172.6 (9.3)	173.8 (9.7)[a]	74.8 (14.0)	78.2 (14.8)[a]	13.7	12.3
1998-1999	6 543	45.3	1 577	44.6	42.0 (10.3)	44.1 (10.8)[a]	173.9 (9.1)	174.0 (9.4)	76.1 (14.0)	77.9 (15.2)[a]	19.1	19.5
2000-2001	12 545	49.5	3 370	48.4	42.3 (10.7)	45.4 (11.2)[a]	173.3 (9.2)	173.3 (9.5)	76.0 (14.2)	79.1 (15.6)[a]	20.8	19.4
2002-2003	22 629	52.4	5 167	53.3	41.9 (11.2)	47.3 (11.1)[a]	172.8 (9.2)	172.4 (9.6)[a]	75.4 (14.4)	79.0 (16.4)[a]	19.9	17.7[a]
2004-2005	37 420	52.1	9 704	48.7[a]	42.8 (10.9)	47.5 (11.1)[a]	173.0 (9.2)	173.3 (9.5)[a]	75.9 (14.6)	80.0 (16.4)[a]	25.3	20.5[a]
2006-2007	38 519	48.6	9 703	46.0[a]	42.9 (11.1)	47.1 (11.4)[a]	173.5 (9.3)	173.6 (9.7)	77.1 (15.0)	80.5 (17.0)[a]	24.5	20.3[a]
2008-2009	43 479	46.2	9 672	47.8[a]	42.8 (11.3)	46.7 (12.0)[a]	173.9 (9.3)	173.3 (9.7)[a]	77.9 (15.3)	80.6 (17.9)[a]	25.9	22.5[a]
2010-2011	39 177	44.2	7 758	45.6[a]	42.4 (11.2)	46.0 (12.0)[a]	174.3 (9.4)	173.8 (9.7)[a]	78.7 (15.5)	82.0 (18.1)[a]	27.3	22.9[a]
2012-2013	57 246	40.8	12 135	40.6	42.1 (11.3)	46.2 (11.7)[a]	174.9 (9.3)	174.9 (9.7)	79.0 (15.4)	82.8 (17.9)[a]	31.5	26.2[a]
2014-2015	55 584	37.6	13 070	38.2	41.7 (11.5)	45.0 (12.3)[a]	175.3 (9.2)	175.2 (9.8)	79.9 (15.7)	83.2 (18.6)[a]	29.7	24.6[a]
2016-2017	36 561	36.8	8 878	37.9	41.1 (11.7)	43.9 (12.4)[a]	175.4 (9.3)	175.6 (9.6)	80.3 (16.0)	83.3 (18.4)[a]	29.5	26.5[a]
Total	354 277	44.2	81 849	44.0	42.2 (11.2)	46.0 (11.8)[a]	174.2 (9.3)	174.1 (9.7)[a]	78.1 (15.3)	81.4 (17.4)[a]	26.9	22.8[a]

[a] Different from individuals with VO_2max during the same time period ($p>0.05$) using chi^2-test for percentages, independent t-test for age and general linear modelling for height and weight.

[#] Mean and SE values adjusted for age and gender

SD, standard deviation

SE, standard error
