# Supplementary material for: Decline in cardiorespiratory fitness in the Swedish working force between 1995 and 2017
Source: Scand J Med Sci Sports. 2018 Nov 15;29(2):232–9. doi: 10.1111/sms.13328 (PMC7379642; doi:10.1111/sms.13328)
Supplement: Supplementary file 2 [file SMS-29-232-s002.pdf]

Supplement Table 2. Change in VO_2max ($L{\cdot}min^{-1}$ and $ml{\cdot}min^{-1}{\cdot}kg^{-1}$) from 1995-1997 to 2016-2017 in the total population and by gender.

	Women				Men				Total			
	**$L{\cdot}min^{-1}$**		**$ml{\cdot}min^{-1}{\cdot}kg^{-1}$**		**$L{\cdot}min^{-1}$**		**$ml{\cdot}min^{-1}{\cdot}kg^{-1}$**			**$L{\cdot}min^{-1}$**		**$ml{\cdot}min^{-1}{\cdot}kg^{-1}$**
Year	Mean (SD)	Change	Mean (SD)	Change	Mean (SD)	Change	Mean (SD)	Change	Mean (SD)	Change	Mean (SD)	Change
95-97	2.47 (0.07)	Ref	38.1 (1.27)	Ref	3.16 (0.09)	Ref	39.0 (1.22)	Ref	2.80 (0.08)	Ref	38.5 (0.90)	Ref
98-99	2.42 (0.09)	-2,1%	36.8 (1.40)	-3,4%	3.05 (0.10)	-3,4%	37.5 (1.45)	-3,9%	2.74 (0.09)	-2,2%	37.1 (1.00)	-3,6%
00-01	2.45 (0.08)	-1,0%	36.9 (1.33)	-3,2%	3.04 (0.10)	-3,9%	36.7 (1.49)	-5,9%	2.75 (0.08)	-1,8%	36.8 (0.99)	-4,4%
02-03	2.33 (0.08)	-5,8%	35.2 (1.28)	-7,5%	2.94 (0.10)	-6,9%	35.9 (1.39)	-8,0%	2.64 (0.08)	-5,8%	35.6 (0.94)	-7,6%
04-05	2.35 (0.07)	-4,9%	35.2 (1.32)	-7,7%	2.97 (0.09)	-6,1%	36.4 (1.16)	-6,6%	2.66 (0.08)	-4,9%	35.8 (0.87)	-7,0%
06-07	2.37 (0.07)	-4,1%	35.4 (1.22)	-7,1%	2.97 (0.09)	-6,1%	35.9 (1.15)	-8,0%	2.67 (0.07)	-4,6%	35.6 (0.83)	-7,4%
08-09	2.39 (0.07)	-3,1%	35.5 (1.19)	-6,8%	2.97 (0.09)	-6,1%	35.7 (1.21)	-8,5%	2.68 (0.07)	-4,2%	35.6 (0.84)	-7,5%
10-11	2.38 (0.07)	-3,5%	35.1 (1.25)	-7,8%	2.99 (0.08)	-5,5%	35.7 (1.11)	-8,5%	2.69 (0.07)	-4,0%	35.4 (0.83)	-8,1%
12-13	2.38 (0.07)	-3,8%	35.0 (1.21)	-8,2%	2.92 (0.09)	-7,5%	35.0 (1.18)	-10,3%	2.65 (0.07)	-5,3%	35.0 (0.83)	-9,1%
14-15	2.34 (0.07)	-5,2%	34.4 (1.17)	-9,7%	2.90 (0.08)	-8,4%	34.4 (1.08)	-11,7%	2.62 (0.07)	-6,4%	34.4 (0.79)	-10,6%
16-17	2.34 (0.07)	-5,3%	34.5 (1.12)	-9,4%	2.88 (0.08)	-8,7%	34.2 (1.09)	-12,4%	2.61 (0.07)	-6,7%	34.3 (0.77)	-10,8%
