# Supplementary material for: Decline in cardiorespiratory fitness in the Swedish working force between 1995 and 2017
Source: Scand J Med Sci Sports. 2018 Nov 15;29(2):232–9. doi: 10.1111/sms.13328 (PMC7379642; doi:10.1111/sms.13328)
Supplement: Supplementary file 3 [file SMS-29-232-s003.pdf]

Supplement Table 3. Change in VO_2max ($L \cdot min^{-1}$ and $ml \cdot min^{-1} \cdot kg^{-1}$) from 1995-1997 to 2016-2017 in the total population and by age-group.

		**18-34 years**					**35-49 years**					**50-74 years**			
		**$L \cdot min^{-1}$**		**$ml \cdot min^{-1} \cdot kg^{-1}$**			**$L \cdot min^{-1}$**		**$ml \cdot min^{-1} \cdot kg^{-1}$**				**$L \cdot min^{-1}$**		**$ml \cdot min^{-1} \cdot kg^{-1}$**
Year	n	Mean (SD)	Change	Mean (SD)	Change	n	Mean (SD)	Change	Mean (SD)	Change	n	Mean (SD)	Change	Mean (SD)	Change
95-97	1 354	3.19 (0.17)	Ref	43.9 (0.72)	Ref	2 195	2.82 (0.16)	Ref	38.0 (0.52)	Ref	1 025	2.44 (0.14)	Ref	32.5 (0.68)	Ref
98-99	1 840	3.19 (0.16)	-0,1%	43.7 (0.64)	-0,5%	2 849	2.80 (0.14)	-0,6%	37.5 (0.39)	-1,4%	1 854	2.42 (0.12)	-0,7%	32.3 (0.51)	-0,7%
00-01	3 469	3.16 (0.16)	-1,0%	43.0 (0.76)	-2,0%	5 248	2.82 (0.14)	-0,1%	37.5 (0.70)	-1,3%	3 828	2.40 (0.13)	-1,7%	31.8 (0.56)	-2,1%
02-03	6 563	3.09 (0.17)	-3,3%	42.3 (0.68)	-3,7%	9 429	2.78 (0.15)	-1,4%	36.8 (0.70)	-3,1%	6 637	2.35 (0.13)	-3,7%	31.2 (0.60)	-3,9%
04-05	9 617	3.09 (0.16)	-3,0%	42.2 (0.57)	-3,9%	16 294	2.78 (0.15)	-1,3%	36.7 (0.69)	-3,5%	11 509	2.35 (0.13)	-3,6%	31.1 (0.50)	-4,4%
06-07	9 743	3.08 (0.15)	-3,4%	41.7 (0.61)	-4,9%	16 867	2.79 (0.15)	-0,9%	36.5 (0.69)	-3,9%	11 909	2.37 (0.13)	-2,9%	31.2 (0.56)	-4,1%
08-09	11 268	3.07 (0.15)	-3,7%	41.6 (0.70)	-5,2%	18 652	2.82 (0.15)	-0,1%	36.6 (0.87)	-3,6%	13 559	2.39 (0.12)	-2,0%	31.2 (0.59)	-4,1%
10-11	10 340	3.06 (0.15)	-4,0%	41.3 (0.78)	-5,8%	17 618	2.83 (0.15)	0,2%	36.5 (0.86)	-3,8%	11 219	2.41 (0.13)	-1,2%	31.1 (0.58)	-4,2%
12-13	15 737	3.04 (0.14)	-4,7%	41.0 (0.82)	-6,7%	25 651	2.79 (0.14)	-1,1%	36.2 (0.94)	-4,7%	15 858	2.38 (0.12)	-2,3%	30.8 (0.56)	-5,2%
14-15	16 428	2.99 (0.14)	-6,4%	40.1 (0.67)	-8,5%	23 725	2.75 (0.13)	-2,5%	35.6 (0.90)	-6,4%	15 431	2.37 (0.12)	-2,7%	30.5 (0.65)	-6,2%
16-17	11 944	2.98 (0.14)	-6,5%	39.9 (0.77)	-9,2%	14 693	2.73 (0.13)	-3,2%	35.3 (0.84)	-7,1%	9 924	2.38 (0.13)	-2,3%	30.5 (0.71)	-6,1%
