# Supplementary material for: Decline in cardiorespiratory fitness in the Swedish working force between 1995 and 2017
Source: Scand J Med Sci Sports. 2018 Nov 15;29(2):232–9. doi: 10.1111/sms.13328 (PMC7379642; doi:10.1111/sms.13328)
Supplement: Supplementary file 4 [file SMS-29-232-s004.pdf]

Supplement Table 4. Change in VO_2max (ml·min^{-1}·kg^{-1}) from 1995-1997 to 2016-2017 in relation to length of education.

			**≤9 years**					**10-12 years**					**≥12 years**		
		**L·min^{-1}**		**ml·min^{-1}·kg^{-1}**			**L·min^{-1}**		**ml·min^{-1}·kg^{-1}**				**L·min^{-1}**		**ml·min^{-1}·kg^{-1}**
Year	n	Mean (SD)	Change	Mean (SD)	Change	n	Mean (SD)	Change	Mean (SD)	Change	n	Mean (SD)	Change	Mean (SD)	Change
95-97	731	2.74 (0.12)	Ref	36.2 (1.30)	Ref	3 216	2.80 (0.15)	Ref	38.5 (1.55)	Ref	627	2.84 (0.13)	Ref	39.9 (1.45)	Ref
98-99	880	2.64 (0.14)	-3,5%	34.5 (2.09)	-4,6%	4 416	2.72 (0.16)	-2,7%	37.1 (1.67)	-3,7%	1 247	2.83 (0.13)	-0,2%	39.0 (1.64)	-2,2%
00-01	1 543	2.63 (0.12)	-4,2%	35.0 (1.50)	-3,3%	8 398	2.80 (0.13)	-0,1%	36.9 (1.59)	-4,2%	2 604	2.71 (0.17)	-4,5%	37.6 (2.19)	-5,7%
02-03	2 572	2.43 (0.15)	-11,5%	32.5 (1.84)	-10,3%	15 551	2.66 (0.14)	-5,0%	35.5 (1.56)	-7,8%	4 506	2.74 (0.13)	-3,6%	37.9 (1.51)	-4,9%
04-05	3 625	2.55 (0.14)	-7,1%	33.8 (1.68)	-6,6%	24 312	2.67 (0.13)	-4,7%	35.6 (1.46)	-7,5%	9 483	2.72 (0.13)	-4,1%	37.8 (1.52)	-5,3%
06-07	3 909	2.57 (0.14)	-6,3%	33.5 (1.60)	-7,4%	25 167	2.67 (0.13)	-4,7%	35.4 (1.39)	-8,1%	9 443	2.75 (0.13)	-3,3%	37.9 (1.36)	-4,9%
08-09	4 171	2.51 (0.12)	-8,2%	32.7 (1.64)	-9,6%	28 057	2.69 (0.13)	-3,9%	35.4 (1.33)	-8,1%	11 251	2.78 (0.13)	-2,1%	38.3 (1.43)	-4,0%
10-11	3 626	2.55 (0.12)	-7,0%	32.9 (1.38)	-9,2%	24 837	2.68 (0.13)	-4,2%	35.0 (1.38)	-9,1%	10 714	2.80 (0.13)	-1,5%	38.3 (1.41)	-4,1%
12-13	4 384	2.47 (0.11)	-9,7%	31.9 (1.42)	-11,8%	34 838	2.67 (0.12)	-4,8%	34.7 (1.35)	-9,8%	18 024	2.75 (0.13)	-3,3%	37.9 (1.46)	-5,0%
14-15	4 053	2.45 (0.12)	-10,6%	31.5 (1.41)	-13,1%	35 047	2.63 (0.12)	-5,9%	34.2 (1.26)	-11,2%	16 484	2.71 (0.12)	-4,6%	37.2 (1.34)	-6,7%
16-17	2 446	2.43 (0.12)	-11,4%	31.6 (1.22)	-12,8%	23 341	2.63 (0.12)	-6,2%	34.1 (1.30)	-11,5%	10 774	2.71 (0.12)	-4,5%	37.1 (1.27)	-7,0%
