# Supplementary material for: Decline in cardiorespiratory fitness in the Swedish working force between 1995 and 2017
Source: Scand J Med Sci Sports. 2018 Nov 15;29(2):232–9. doi: 10.1111/sms.13328 (PMC7379642; doi:10.1111/sms.13328)
Supplement: Supplementary file 5 [file SMS-29-232-s005.pdf]

Supplement Table 5. Change in VO_2max ($ml \cdot min^{-1} \cdot kg^{-1}$) from 1995-1997 to 2016-2017 in relation to region.

	**Urban counties**					**Rural counties**					**All other counties**				
		**L·min⁻¹**		**ml·min⁻¹·kg⁻¹**			**L·min⁻¹**		**ml·min⁻¹·kg⁻¹**			**L·min⁻¹**		**ml·min⁻¹·kg⁻¹**	
Year	n	Mean (SD)	Change	Mean (SD)	Change	n	Mean (SD)	Change	Mean (SD)	Change	n	Mean (SD)	Change	Mean (SD)	Change
95-97	882	2.72 (0.05)	Ref	37.4 (0.60)	Ref	2241	2.82 (0.05)	Ref	38.2 (0.54)	Ref	1451	2.88 (0.05)	Ref	39.7 (0.57)	Ref
98-99	2017	2.72 (0.06)	0,2%	37.3 (0.68)	-0,3%	2565	2.86 (0.05)	1,4%	38.3 (0.53)	0,4%	1961	2.78 (0.04)	-3,5%	37.7 (0.46)	-5,1%
00-01	4629	2.76 (0.05)	1,6%	37.2 (0.62)	-0,5%	3171	2.83 (0.04)	0,2%	37.5 (0.48)	-1,9%	4744	2.78 (0.04)	-3,5%	37.6 (0.50)	-5,3%
02-03	9973	2.62 (0.05)	-3,6%	35.5 (0.54)	-5,1%	5659	2.70 (0.04)	-4,2%	35.6 (0.56)	-6,8%	6996	2.73 (0.04)	-5,4%	36.8 (0.53)	-7,4%
04-05	15177	2.64 (0.04)	-2,8%	35.3 (0.54)	-5,8%	9660	2.66 (0.04)	-5,9%	35.5 (0.47)	-6,9%	12580	2.71 (0.04)	-6,0%	36.9 (0.46)	-7,0%
06-07	17130	2.65 (0.04)	-2,4%	35.6 (0.47)	-4,9%	8053	2.68 (0.05)	-5,0%	35.3 (0.48)	-7,6%	13332	2.68 (0.04)	-6,8%	35.9 (0.50)	-9,6%
08-09	22928	2.69 (0.04)	-1,0%	35.9 (0.47)	-4,1%	8028	2.66 (0.04)	-5,6%	34.5 (0.51)	-9,6%	12519	2.68 (0.04)	-7,0%	35.5 (0.50)	-10,5%
10-11	20374	2.69 (0.04)	-0,9%	35.6 (0.48)	-4,9%	6129	2.70 (0.04)	-4,3%	35.5 (0.42)	-7,1%	12669	2.68 (0.04)	-7,0%	35.2 (0.48)	-11,4%
12-13	31735	2.64 (0.04)	-2,9%	35.0 (0.49)	-6,5%	7736	2.68 (0.04)	-4,9%	34.7 (0.43)	-9,1%	17768	2.66 (0.04)	-7,6%	35.0 (0.49)	-11,8%
14-15	28977	2.60 (0.04)	-4,2%	34.4 (0.45)	-8,1%	9383	2.64 (0.04)	-6,3%	34.2 (0.43)	-10,5%	17220	2.65 (0.04)	-8,1%	34.6 (0.45)	-12,8%
16-17	13652	2.62 (0.03)	-3,5%	34.5 (0.40)	-7,8%	4589	2.64 (0.03)	-6,5%	34.2 (0.40)	-10,5%	7317	2.61 (0.04)	-9,4%	34.2 (0.39)	-14,0%
