# Supplementary material for: Decline in cardiorespiratory fitness in the Swedish working force between 1995 and 2017
Source: Scand J Med Sci Sports. 2018 Nov 15;29(2):232–9. doi: 10.1111/sms.13328 (PMC7379642; doi:10.1111/sms.13328)
Supplement: Supplementary file 6 [file SMS-29-232-s006.pdf]

Supplement Table 6. Change in VO_2max ($L \cdot min^{-1}$ and $ml \cdot min^{-1} \cdot kg^{-1}$) from 1995-1997 to 2016-2017 in relation to sex and age.

Women

	18-34 years					35-49 years					50-74 years				
		L·min⁻¹		ml·min⁻¹·kg⁻¹			L·min⁻¹		ml·min⁻¹·kg⁻¹			L·min⁻¹		ml·min⁻¹·kg⁻¹	
Year	n	Mean (SD)	Change	Mean (SD)	Change	n	Mean (SD)	Change	Mean (SD)	Change	n	Mean (SD)	Change	Mean (SD)	Change
95-97	646	2.82 (0.05)	Ref	43.8 (1.09)	Ref	1 160	2.47 (0.03)	Ref	37.4 (0.84)	Ref	589	2.13 (0.05)	Ref	31.5 (1.03)	Ref
98-99	767	2.82 (0.05)	-0,1%	43.1 (1.14)	-1,6%	1 302	2.50 (0.02)	1,2%	37.5 (0.53)	0,3%	895	2.16 (0.04)	1,3%	31.9 (0.81)	1,4%
00-01	1 626	2.80 (0.06)	-0,5%	42.6 (1.26)	-2,7%	2 592	2.51 (0.05)	1,7%	37.6 (1.11)	0,6%	1 988	2.13 (0.02)	-0,2%	31.5 (0.71)	-0,1%
02-03	2 897	2.72 (0.05)	-3,7%	41.7 (1.01)	-4,7%	5 026	2.45 (0.04)	-1,0%	36.5 (1.03)	-2,4%	3 935	2.06 (0.04)	-3,3%	30.5 (0.79)	-3,3%
04-05	4 397	2.73 (0.04)	-3,2%	41.9 (0.95)	-4,3%	8 724	2.45 (0.04)	-0,6%	36.4 (1.00)	-2,6%	6 379	2.07 (0.03)	-2,7%	30.5 (0.62)	-3,2%
06-07	4 257	2.74 (0.03)	-2,7%	41.8 (0.84)	-4,6%	8 185	2.47 (0.04)	0,2%	36.4 (0.94)	-2,7%	6 272	2.09 (0.05)	-1,8%	30.6 (0.93)	-2,7%
08-09	4 765	2.74 (0.04)	-3,0%	41.6 (1.16)	-5,1%	8 660	2.50 (0.07)	1,3%	36.6 (1.32)	-2,0%	6 643	2.13 (0.05)	-0,1%	30.9 (0.94)	-2,0%
10-11	4 087	2.74 (0.07)	-2,9%	41.4 (1.46)	-5,4%	8 000	2.51 (0.05)	1,7%	36.5 (1.26)	-2,3%	5 214	2.13 (0.05)	0,0%	30.7 (0.81)	-2,5%
12-13	5 727	2.73 (0.06)	-3,3%	41.1 (1.44)	-6,2%	10 823	2.50 (0.05)	1,1%	36.5 (1.33)	-2,4%	6 786	2.13 (0.04)	0,1%	30.6 (0.91)	-2,8%
14-15	5 671	2.68 (0.04)	-5,1%	40.4 (1.00)	-7,8%	9 457	2.46 (0.06)	-0,2%	35.8 (1.42)	-4,2%	5 766	2.12 (0.06)	-0,5%	30.3 (1.09)	-3,8%
16-17	3 743	2.67 (0.07)	-5,3%	40.2 (1.38)	-8,2%	5 904	2.45 (0.05)	-0,9%	35.7 (1.23)	-4,6%	3 817	2.12 (0.07)	-0,6%	30.4 (1.14)	-3,6%

Men

	18-34 years					35-49 years					50-74 years				
		L·min⁻¹		ml·min⁻¹·kg⁻¹			L·min⁻¹		ml·min⁻¹·kg⁻¹			L·min⁻¹		ml·min⁻¹·kg⁻¹	
Year	n	Mean (SD)	Change	Mean (SD)	Change	n	Mean (SD)	Change	Mean (SD)	Change	n	Mean (SD)	Change	Mean (SD)	Change
95-97	708	3.54 (0.08)	Ref	43.9 (1.19)	Ref	1 035	3.16 (0.03)	Ref	38.5 (0.59)	Ref	436	2.74 (0.03)	Ref	33.5 (0.42)	Ref
98-99	1 073	3.54 (0.05)	-0,1%	44.3 (0.65)	0,8%	1 547	3.10 (0.04)	-1,9%	37.4 (0.69)	-2,8%	959	2.69 (0.04)	-1,8%	32.6 (0.72)	-2,7%
00-01	1 843	3.49 (0.09)	-1,3%	43.4 (1.09)	-1,2%	2 656	3.12 (0.06)	-1,4%	37.4 (1.10)	-2,9%	1 840	2.67 (0.07)	-2,5%	32.2 (0.97)	-4,0%
02-03	3 666	3.44 (0.08)	-2,9%	42.7 (1.03)	-2,6%	4 403	3.11 (0.07)	-1,7%	37.2 (1.14)	-3,5%	2 702	2.64 (0.06)	-3,6%	32.0 (0.79)	-4,6%
04-05	5 220	3.44 (0.05)	-2,8%	42.4 (0.82)	-3,4%	7 570	3.10 (0.07)	-1,8%	36.9 (1.15)	-4,1%	5 130	2.63 (0.04)	-4,0%	31.7 (0.73)	-5,5%
06-07	5 486	3.40 (0.06)	-3,9%	41.7 (1.06)	-5,1%	8 682	3.11 (0.07)	-1,7%	36.6 (1.21)	-4,9%	5 637	2.65 (0.05)	-3,3%	31.7 (0.65)	-5,3%
08-09	6 503	3.40 (0.07)	-4,1%	41.7 (1.07)	-5,1%	9 992	3.13 (0.08)	-1,0%	36.6 (1.42)	-5,0%	6 916	2.66 (0.06)	-3,0%	31.5 (0.89)	-6,0%
10-11	6 253	3.37 (0.07)	-4,8%	41.2 (0.96)	-6,0%	9 618	3.13 (0.08)	-0,8%	36.6 (1.46)	-5,0%	6 005	2.69 (0.06)	-1,8%	31.6 (0.92)	-5,8%
12-13	10 010	3.34 (0.07)	-5,6%	40.8 (1.15)	-7,0%	14 828	3.07 (0.09)	-2,7%	36.0 (1.59)	-6,6%	9 072	2.64 (0.06)	-3,8%	31.0 (0.84)	-7,5%
14-15	10 757	3.28 (0.07)	-7,3%	39.9 (1.10)	-9,0%	14 268	3.03 (0.06)	-4,2%	35.3 (1.39)	-8,4%	9 665	2.63 (0.07)	-4,1%	30.7 (0.96)	-8,5%
16-17	8 201	3.28 (0.06)	-7,3%	39.6 (0.99)	-9,9%	8 789	3.01 (0.07)	-4,9%	34.9 (1.38)	-9,2%	6 107	2.65 (0.08)	-3,2%	30.6 (1.10)	-8,5%
