# Supplementary material for: Decline in cardiorespiratory fitness in the Swedish working force between 1995 and 2017
Source: Scand J Med Sci Sports. 2018 Nov 15;29(2):232–9. doi: 10.1111/sms.13328 (PMC7379642; doi:10.1111/sms.13328)
Supplement: Supplementary file 7 [file SMS-29-232-s007.pdf]

Supplement Table 7. Change in VO_2max ($L \cdot min^{-1}$ and $ml \cdot min^{-1} \cdot kg^{-1}$) from 1995-1997 to 2016-2017 in relation to sex and length of education.

Women

	**≤9 years**					**10-12 years**					**≥12 years**				
		**$L \cdot min^{-1}$**		**$ml \cdot min^{-1} \cdot kg^{-1}$**			**$L \cdot min^{-1}$**		**$ml \cdot min^{-1} \cdot kg^{-1}$**			**$L \cdot min^{-1}$**		**$ml \cdot min^{-1} \cdot kg^{-1}$**	
Year	n	Mean (SD)	Change	Mean (SD)	Change	n	Mean (SD)	Change	Mean (SD)	Change	n	Mean (SD)	Change	Mean (SD)	Change
95-97	278	2.41 (0.12)	Ref	36.1 (2.42)	Ref	1 719	2.44 (0.12)	Ref	37.6 (2.18)	Ref	398	2.59 (0.12)	Ref	40.2 (2.16)	Ref
98-99	324	2.44 (0.15)	1,4%	36.5 (2.65)	1,2%	1 998	2.35 (0.17)	-3,9%	35.7 (2.39)	-5,0%	642	2.59 (0.14)	0,1%	39.6 (2.37)	-1,5%
00-01	721	2.33 (0.10)	-3,2%	33.9 (1.99)	-6,0%	4 172	2.47 (0.14)	1,2%	37.2 (2.33)	-1,0%	1 313	2.46 (0.19)	-5,0%	37.9 (2.74)	-5,6%
02-03	1 154	2.12 (0.17)	-12,1%	32.1 (2.54)	-11,1%	8 157	2.33 (0.13)	-4,7%	35.0 (2.18)	-6,9%	2 547	2.45 (0.12)	-5,5%	37.4 (2.20)	-6,8%
04-05	1 512	2.17 (0.16)	-9,9%	32.6 (2.60)	-9,7%	12 670	2.34 (0.13)	-4,1%	34.7 (2.30)	-7,7%	5 318	2.46 (0.13)	-4,9%	37.6 (2.20)	-6,3%
06-07	1 546	2.18 (0.16)	-9,6%	32.5 (2.62)	-10,0%	12 075	2.36 (0.12)	-3,3%	35.0 (2.09)	-6,8%	5 093	2.49 (0.11)	-3,8%	37.8 (1.89)	-6,0%
08-09	1 416	2.26 (0.13)	-6,1%	33.0 (2.27)	-8,5%	12 591	2.37 (0.12)	-3,0%	34.9 (1.98)	-7,1%	6 061	2.52 (0.12)	-2,6%	38.2 (2.06)	-4,9%
10-11	1 131	2.22 (0.12)	-7,8%	32.3 (1.98)	-10,5%	10 401	2.36 (0.13)	-3,2%	34.5 (2.13)	-8,2%	5 769	2.52 (0.13)	-2,8%	38.0 (2.24)	-5,4%
12-13	1 212	2.23 (0.10)	-7,4%	32.1 (1.89)	-11,2%	13 049	2.36 (0.12)	-3,3%	34.4 (2.03)	-8,4%	9 075	2.49 (0.13)	-3,7%	37.9 (2.22)	-5,8%
14-15	982	2.15 (0.13)	-11,0%	30.8 (2.21)	-14,7%	11 722	2.33 (0.12)	-4,4%	34.0 (1.94)	-9,6%	8 190	2.47 (0.11)	-4,8%	37.3 (1.88)	-7,2%
16-17	521	2.14 (0.10)	-11,1%	31.4 (1.44)	-13,1%	7 351	2.32 (0.13)	-4,7%	34.0 (2.04)	-9,6%	5 592	2.48 (0.11)	-4,3%	37.4 (1.71)	-6,9%

Men

	**≤9 years**					**10-12 years**					**≥12 years**				
		**$L \cdot min^{-1}$**		**$ml \cdot min^{-1} \cdot kg^{-1}$**			**$L \cdot min^{-1}$**		**$ml \cdot min^{-1} \cdot kg^{-1}$**			**$L \cdot min^{-1}$**		**$ml \cdot min^{-1} \cdot kg^{-1}$**	
Year	n	Mean (SD)	Change	Mean (SD)	Change	n	Mean (SD)	Change	Mean (SD)	Change	n	Mean (SD)	Change	Mean (SD)	Change
95-97	453	2.98 (0.12)	Ref	36.3 (1.44)	Ref	1 497	3.19 (0.17)	Ref	39.5 (2.33)	Ref	180	3.21 (0.17)	Ref	39.5 (2.08)	Ref
98-99	556	2.75 (0.20)	-7,6%	33.4 (3.12)	-8,0%	2 418	3.13 (0.17)	-2,0%	38.5 (2.39)	-2,5%	605	3.13 (0.17)	-2,6%	38.3 (2.46)	-2,9%
00-01	822	2.93 (0.15)	-1,6%	36.2 (2.35)	-0,4%	4 226	3.05 (0.15)	-4,4%	36.7 (2.33)	-7,2%	1 291	3.06 (0.24)	-4,8%	37.2 (3.78)	-5,9%
02-03	1 418	2.66 (0.20)	-10,6%	32.7 (2.81)	-9,8%	7 394	2.97 (0.18)	-7,0%	36.0 (2.38)	-8,9%	1 959	3.14 (0.16)	-2,3%	38.6 (2.23)	-2,3%
04-05	2 113	2.84 (0.16)	-4,8%	34.8 (2.29)	-4,3%	11 642	2.97 (0.16)	-6,8%	36.4 (1.93)	-7,7%	4 165	3.08 (0.18)	-4,1%	38.0 (2.29)	-3,8%
06-07	2 363	2.87 (0.14)	-3,7%	34.3 (2.08)	-5,5%	13 092	2.96 (0.16)	-7,4%	35.7 (1.99)	-9,7%	4 350	3.10 (0.17)	-3,6%	38.1 (2.14)	-3,5%
08-09	2 755	2.71 (0.18)	-9,1%	32.5 (2.51)	-10,5%	15 466	2.99 (0.15)	-6,2%	35.8 (1.93)	-9,4%	5 190	3.14 (0.17)	-2,3%	38.4 (2.15)	-2,8%
10-11	2 495	2.80 (0.14)	-6,0%	33.3 (2.06)	-8,2%	14 436	2.98 (0.15)	-6,6%	35.5 (1.90)	-10,2%	4 945	3.18 (0.14)	-0,9%	38.6 (1.83)	-2,3%
12-13	3 172	2.66 (0.17)	-10,7%	31.8 (2.23)	-12,3%	21 789	2.95 (0.15)	-7,6%	35.0 (1.94)	-11,4%	8 949	3.10 (0.15)	-3,6%	37.9 (2.05)	-3,9%
14-15	3 071	2.69 (0.15)	-9,8%	32.0 (1.93)	-11,9%	23 325	2.91 (0.13)	-8,7%	34.4 (1.77)	-13,0%	8 294	3.04 (0.15)	-5,3%	37.1 (2.09)	-6,0%
16-17	1 925	2.65 (0.16)	-11,1%	31.7 (2.03)	-12,6%	15 990	2.91 (0.14)	-8,9%	34.1 (1.78)	-13,6%	5 182	3.03 (0.16)	-5,5%	36.7 (2.06)	-7,1%
