# Supplementary material for: Decline in cardiorespiratory fitness in the Swedish working force between 1995 and 2017
Source: Scand J Med Sci Sports. 2018 Nov 15;29(2):232–9. doi: 10.1111/sms.13328 (PMC7379642; doi:10.1111/sms.13328)
Supplement: Supplementary file 8 [file SMS-29-232-s008.pdf]

Supplement Table 8. Change in VO_2max ($L·min^{-1}$ and $ml·min^{-1}·kg^{-1}$) from 1995-1997 to 2016-2017 in relation to length of education and age-group.

Educational level ≤9 years

	**18-34 years**					**35-49 years**					**50-74 years**				
		**L·min⁻¹**		**ml·min⁻¹·kg⁻¹**			**L·min⁻¹**		**ml·min⁻¹·kg⁻¹**			**L·min⁻¹**		**ml·min⁻¹·kg⁻¹**	
Year	n	Mean (SD)	Change	Mean (SD)	Change	n	Mean (SD)	Change	Mean (SD)	Change	n	Mean (SD)	Change	Mean (SD)	Change
95-97	102	3.05 (0.31)	Ref	41.3 (1.18)	Ref	378	2.75 (0.33)	Ref	35.6 (0.88)	Ref	251	2.41 (0.31)	Ref	31.4 (1.31)	Ref
98-99	111	3.14 (0.33)	3,0%	42.8 (0.69)	3,6%	358	2.71 (0.25)	-1,5%	35.2 (0.26)	-1,1%	411	2.37 (0.27)	-1,7%	30.5 (0.67)	-2,9%
00-01	162	2.99 (0.30)	-2,0%	40.3 (0.83)	-2,4%	567	2.69 (0.31)	-2,2%	34.8 (0.14)	-2,2%	814	2.34 (0.24)	-2,9%	30.2 (0.51)	-3,8%
02-03	438	2.94 (0.34)	-3,6%	40.3 (1.15)	-2,4%	853	2.63 (0.28)	-4,4%	33.7 (0.43)	-5,3%	1 281	2.28 (0.27)	-5,4%	29.6 (0.73)	-5,7%
04-05	509	3.04 (0.35)	-0,3%	41.2 (0.23)	-0,2%	1 189	2.65 (0.27)	-3,6%	33.7 (0.12)	-5,3%	1 927	2.30 (0.27)	-4,6%	29.7 (0.56)	-5,4%
06-07	536	3.05 (0.33)	0,0%	40.5 (0.09)	-1,9%	1 282	2.66 (0.28)	-3,3%	33.5 (0.14)	-5,9%	2 091	2.30 (0.28)	-4,6%	29.5 (0.86)	-6,1%
08-09	659	2.99 (0.28)	-2,0%	40.1 (0.25)	-2,9%	1 262	2.65 (0.32)	-3,6%	33.2 (0.16)	-6,7%	2 250	2.30 (0.26)	-4,6%	29.2 (0.60)	-7,0%
10-11	706	2.93 (0.32)	-3,9%	39.1 (0.70)	-5,3%	1 037	2.71 (0.29)	-1,5%	34.0 (0.24)	-4,5%	1 883	2.34 (0.28)	-2,9%	29.6 (0.52)	-5,7%
12-13	929	2.90 (0.30)	-4,9%	38.2 (0.41)	-7,5%	1 289	2.62 (0.23)	-4,7%	32.9 (0.60)	-7,6%	2 166	2.29 (0.24)	-5,0%	29.0 (0.30)	-7,6%
14-15	982	2.89 (0.28)	-5,2%	38.2 (0.04)	-7,5%	1 100	2.62 (0.31)	-4,7%	32.6 (0.38)	-8,4%	1 971	2.25 (0.26)	-6,6%	28.4 (0.52)	-9,6%
16-17	746	2.85 (0.34)	-6,6%	37.0 (0.77)	-10,4%	738	2.59 (0.25)	-5,8%	32.4 (0.16)	-9,0%	962	2.26 (0.28)	-6,2%	28.5 (0.51)	-9,2%

Educational level 10-12 years

	**18-34 years**					**35-49 years**					**50-74 years**				
		**L·min⁻¹**		**ml·min⁻¹·kg⁻¹**			**L·min⁻¹**		**ml·min⁻¹·kg⁻¹**			**L·min⁻¹**		**ml·min⁻¹·kg⁻¹**	
Year	n	Mean (SD)	Change	Mean (SD)	Change	n	Mean (SD)	Change	Mean (SD)	Change	n	Mean (SD)	Change	Mean (SD)	Change
95-97	1 136	3.21 (0.38)	Ref	43.9 (0.83)	Ref	1 513	2.85 (0.36)	Ref	37.8 (0.82)	Ref	567	2.42 (0.31)	Ref	32.3 (1.31)	Ref
98-99	1 434	3.18 (0.38)	-0,9%	43.2 (0.89)	-1,6%	1 925	2.83 (0.31)	-0,7%	37.4 (0.03)	-1,1%	1 057	2.42 (0.27)	0,0%	32.4 (0.31)	0,3%
00-01	2 446	3.18 (0.36)	-0,9%	42.9 (0.48)	-2,3%	3 642	2.82 (0.30)	-1,1%	36.8 (0.01)	-2,6%	2 310	2.39 (0.27)	-1,2%	31.8 (0.20)	-1,5%
02-03	4 726	3.11 (0.36)	-3,1%	42.0 (0.40)	-4,3%	6 734	2.79 (0.33)	-2,1%	36.4 (0.42)	-3,7%	4 091	2.35 (0.30)	-2,9%	31.2 (0.88)	-3,4%
04-05	6 059	3.09 (0.36)	-3,7%	41.6 (0.45)	-5,2%	11 248	2.79 (0.33)	-2,1%	36.2 (0.36)	-4,2%	7 005	2.35 (0.28)	-2,9%	31.1 (0.63)	-3,7%
06-07	5 855	3.07 (0.32)	-4,4%	41.2 (0.09)	-6,2%	11 715	2.80 (0.31)	-1,8%	36.0 (0.20)	-4,8%	7 597	2.37 (0.29)	-2,1%	31.2 (0.65)	-3,4%
08-09	6 659	3.07 (0.34)	-4,4%	41.0 (0.34)	-6,6%	12 806	2.81 (0.31)	-1,4%	35.8 (0.10)	-5,3%	8 592	2.40 (0.27)	-0,8%	31.2 (0.29)	-3,4%
10-11	6 058	3.07 (0.32)	-4,4%	40.9 (0.07)	-6,8%	11 612	2.81 (0.31)	-1,4%	35.5 (0.18)	-6,1%	7 167	2.40 (0.28)	-0,8%	31.0 (0.42)	-4,0%
12-13	9 154	3.06 (0.31)	-4,7%	40.6 (0.03)	-7,5%	15 713	2.78 (0.29)	-2,5%	35.2 (0.08)	-6,9%	9 971	2.39 (0.26)	-1,2%	30.8 (0.29)	-4,6%
14-15	10 158	2.99 (0.30)	-6,9%	39.8 (0.20)	-9,3%	14 665	2.74 (0.28)	-3,9%	34.6 (0.18)	-8,5%	10 224	2.38 (0.26)	-1,7%	30.4 (0.22)	-5,9%
16-17	7 732	3.00 (0.30)	-6,5%	39.7 (0.40)	-9,6%	8 856	2.72 (0.28)	-4,6%	34.4 (0.28)	-9,0%	6 753	2.38 (0.27)	-1,7%	30.3 (0.10)	-6,2%

		Educational level >12 years														
	18-34 years					35-49 years					50-74 years				
		$L \cdot min^{-1}$		$ml \cdot min^{-1} \cdot kg^{-1}$			$L \cdot min^{-1}$		$ml \cdot min^{-1} \cdot kg^{-1}$			$L \cdot min^{-1}$		$ml \cdot min^{-1} \cdot kg^{-1}$	
Year	n	Mean (SD)	Change	Mean (SD)	Change	n	Mean (SD)	Change	Mean (SD)	Change	n	Mean (SD)	Change	Mean (SD)	Change
95-97	116	3.22 (0.36)	Ref	45.9 (0.72)	Ref	304	2.78 (0.32)	Ref	39.0 (0.34)	Ref	207	2.53 (0.27)	Ref	34.4 (0.11)	Ref
98-99	295	3.24 (0.36)	0,6%	46.1 (0.21)	0,4%	566	2.79 (0.31)	0,4%	38.3 (0.13)	-1,8%	386	2.49 (0.28)	-1,6%	33.9 (0.46)	-1,5%
00-01	861	3.22 (0.38)	0,0%	45.6 (0.58)	-0,7%	1 039	2.86 (0.32)	2,9%	39.7 (0.18)	1,8%	704	2.48 (0.34)	-2,0%	33.8 (1.07)	-1,7%
02-03	1 399	3.12 (0.43)	-3,1%	44.6 (1.12)	-2,8%	1 842	2.80 (0.36)	0,7%	38.7 (0.68)	-0,8%	1 265	2.42 (0.30)	-4,3%	33.1 (0.72)	-3,8%
04-05	3 049	3.13 (0.38)	-2,8%	44.6 (0.40)	-2,8%	3 857	2.81 (0.35)	1,1%	38.6 (0.60)	-1,0%	2 577	2.40 (0.30)	-5,1%	32.6 (0.78)	-5,2%
06-07	3 352	3.13 (0.38)	-2,8%	44.3 (0.64)	-3,5%	3 870	2.83 (0.35)	1,8%	38.5 (0.61)	-1,3%	2 221	2.44 (0.27)	-3,6%	32.9 (0.19)	-4,4%
08-09	3 950	3.14 (0.37)	-2,5%	44.6 (0.25)	-2,8%	4 584	2.89 (0.34)	4,0%	39.2 (0.44)	0,5%	2 717	2.48 (0.29)	-2,0%	33.3 (0.46)	-3,2%
10-11	3 576	3.14 (0.34)	-2,5%	44.5 (0.23)	-3,1%	4 969	2.89 (0.35)	4,0%	39.3 (0.56)	0,8%	2 169	2.51 (0.30)	-0,8%	33.4 (0.74)	-2,9%
12-13	5 654	3.10 (0.34)	-3,7%	44.2 (0.02)	-3,7%	8 649	2.86 (0.33)	2,9%	39.2 (0.34)	0,5%	3 721	2.47 (0.27)	-2,4%	32.9 (0.13)	-4,4%
14-15	5 288	3.03 (0.35)	-5,9%	42.9 (0.36)	-6,5%	7 960	2.81 (0.31)	1,1%	38.4 (0.14)	-1,5%	3 236	2.48 (0.27)	-2,0%	32.9 (0.19)	-4,4%
16-17	3 466	3.04 (0.34)	-5,6%	42.6 (0.08)	-7,2%	5 099	2.79 (0.31)	0,4%	38.0 (0.16)	-2,6%	2 209	2.53 (0.29)	0,0%	33.4 (0.38)	-2,9%
