# Supplementary material for: Decline in cardiorespiratory fitness in the Swedish working force between 1995 and 2017
Source: Scand J Med Sci Sports. 2018 Nov 15;29(2):232–9. doi: 10.1111/sms.13328 (PMC7379642; doi:10.1111/sms.13328)
Supplement: Supplementary file 9 [file SMS-29-232-s009.pdf]

Supplement Table 9. Standardized proportions with a low VO2max (ml·min-1·kg-1) using different cut-offs, in the total population and by sex.

	Women				Men				Total			
Year	**<32 ml**	**<28.5 ml**	**<25 ml**	**<21.5 ml**	**<32 ml**	**<28.5 ml**	**<25 ml**	**<21.5 ml**	**<32 ml**	**<28.5 ml**	**<25 ml**	**<21.5 ml**
95-97	28,3%	16,5%	7,9%	3,6%	26,3%	14,5%	6,2%	2,5%	27,3%	15,6%	7,1%	3,1%
98-99	37,9%	26,9%	8,7%	2,8%	35,1%	20,3%	11,6%	2,3%	36,5%	23,6%	10,2%	2,5%
00-01	36,7%	20,6%	11,3%	3,2%	38,0%	18,3%	9,7%	5,0%	37,4%	19,4%	10,5%	4,1%
02-03	43,7%	27,3%	14,1%	5,5%	39,7%	27,2%	16,3%	4,0%	41,7%	27,2%	15,2%	4,8%
04-05	43,3%	29,1%	15,6%	6,2%	37,5%	23,3%	9,2%	3,7%	40,4%	26,2%	12,4%	5,0%
06-07	41,0%	26,8%	14,4%	4,7%	38,4%	24,3%	12,1%	4,6%	39,7%	25,5%	13,3%	4,7%
08-09	40,3%	25,5%	14,0%	5,8%	40,5%	26,6%	13,7%	4,7%	40,4%	26,1%	13,9%	5,2%
10-11	43,6%	29,1%	16,8%	6,9%	39,4%	26,1%	13,9%	5,1%	41,5%	27,6%	15,3%	6,0%
12-13	43,2%	29,8%	16,7%	7,3%	43,2%	28,5%	15,6%	6,8%	43,2%	29,1%	16,1%	7,0%
14-15	44,9%	31,4%	18,7%	8,5%	45,8%	30,7%	16,4%	7,0%	45,4%	31,0%	17,5%	7,8%
16-17	45,6%	31,8%	17,2%	6,7%	45,5%	31,4%	18,6%	7,6%	45,6%	31,6%	17,9%	7,2%
