# Supplementary material for: Decline in cardiorespiratory fitness in the Swedish working force between 1995 and 2017
Source: Scand J Med Sci Sports. 2018 Nov 15;29(2):232–9. doi: 10.1111/sms.13328 (PMC7379642; doi:10.1111/sms.13328)
Supplement: Supplementary file 10 [file SMS-29-232-s010.pdf]

**Supplement Table 10.** Test for equility of variance in unstandardized mean (SD) of relative $VO_2$max in the first five years (1995-1999) and and the last five years (2013-2017) in relation to sub-groups of sex, age and educational level.

**Women**

| Age-group | Education length | Year | Mean | SD | Levene's Test for Equality of Variances F-value | p-value |
|---|---|---|---|---|---|---|
| 18-34 years | <12 years | 1995-1999 | 42.6 | 9.7 | | |
| | | 2013-2017 | 39.9 | 10.2 | 2.37 | 0.124 |
| | ≥12 years | 1995-1999 | 46.1 | 10.3 | | |
| | | 2013-2017 | 42.8 | 10.2 | 0.38 | 0.538 |
| 35-49 years | <12 years | 1995-1999 | 36.9 | 9.2 | | |
| | | 2013-2017 | 34.8 | 9.2 | 0.06 | 0.803 |
| | ≥12 years | 1995-1999 | 38.4 | 8.8 | | |
| | | 2013-2017 | 38.3 | 9.7 | 8.09 | 0.004 |
| 50-74 years | <12 years | 1995-1999 | 31.2 | 7.8 | | |
| | | 2013-2017 | 30.0 | 7.8 | 1.19 | 0.274 |
| | ≥12 years | 1995-1999 | 33.8 | 7.8 | | |
| | | 2013-2017 | 32.8 | 8.3 | 3.16 | 0.076 |

**Men**

| Age-group | Education length | Year | Mean | SD | Levene's Test for Equality of Variances F-value | p-value |
|---|---|---|---|---|---|---|
| 18-34 years | <12 years | 1995-1999 | 44.1 | 10.3 | | |
| | | 2013-2017 | 39.6 | 9.9 | 6.29 | 0.012 |
| | ≥12 years | 1995-1999 | 46.0 | 10.5 | | |
| | | 2013-2017 | 43.2 | 10.5 | 0.45 | 0.501 |
| 35-49 years | <12 years | 1995-1999 | 37.4 | 8.9 | | |
| | | 2013-2017 | 34.3 | 8.8 | 0.05 | 0.822 |
| | ≥12 years | 1995-1999 | 38.8 | 8.7 | | |
| | | 2013-2017 | 38.7 | 9.6 | 9.78 | 0.002 |
| 50-74 years | <12 years | 1995-1999 | 32.5 | 7.5 | | |
| | | 2013-2017 | 30.3 | 7.6 | 0.46 | 0.499 |
| | ≥12 years | 1995-1999 | 34.4 | 7.5 | | |
| | | 2013-2017 | 33.2 | 8.3 | 4.05 | 0.044 |
